# Supplementary material for: A qualitative exploration of cardiovascular disease patients’ views and experiences with an eHealth cardiac rehabilitation intervention: The PATHway Project
Source: PLoS One. 2020 Jul 6;15(7):e0235274. doi: 10.1371/journal.pone.0235274 (PMC7337342; doi:10.1371/journal.pone.0235274)
Supplement: S1 Table — (DOCX) [file pone.0235274.s001.docx]

| Component  S1 Table Description of the eleven PATHway components | Description | Examples of associated images used as part of debrief |
| --- | --- | --- |
| ExerClass | The Exerclass included dynamic aerobic exercises and resistance exercises. Participant movements, repetition count, energy expenditure and HR were continuously monitored by the Microsoft Band and PATHway proprietary software to provide personalised feedback via a virtual ‘avatar’ coach and to continuously adapt the programme. A motion-capturing component using the Microsoft Kinect sensor assessed participants’ movements during the ExerClass sessions and provided them with feedback on the accuracy of their exercise execution. A Decision Support System [1] using HR (Microsoft Band 2) along with the accuracy derived from the motion capturing component was used to dynamically select exercises during real time. In this context, the goal was to adjust the exercise prescription to achieve the prescribed target HR zone required to maintain and/or improve cardiovascular fitness [2]. Participants could specify the duration of the class themselves (min 10 minutes). The extent to which the participants enjoyed each exercise session was evaluated. The perceptions of effort, strain, discomfort or fatigue experienced during exercise was be measured using the Borg 10 point rating of perceived exertion (RPE) scale. The effect and RPE scores was be used to allow the PATHway platform to automatically adjust the exercise sessions offline according to the participant’s levels of enjoyment and exertion. | 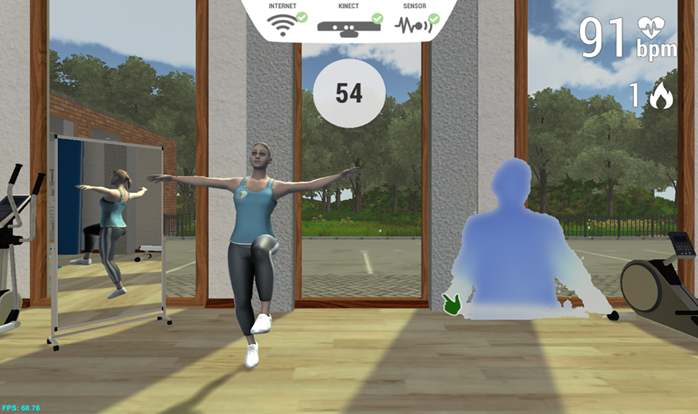 |
| Screening | The screening component evaluated the participants resting HR and blood pressure, medication compliance and eating behaviour prior to engaging with ExerClass. This was utilised to help participants determine whether it was safe for them to engage in exercise. | 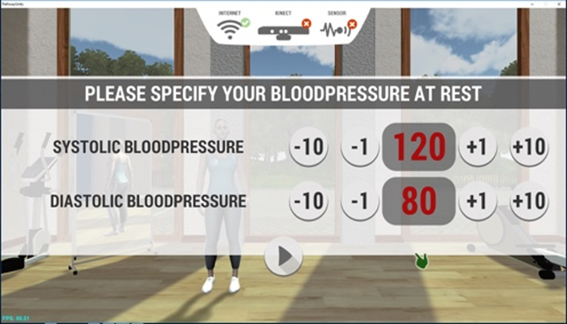 |
| Dashboard | The wrist worn Microsoft band 2 assessed physical activity levels beyond the ExerClasses and ExerGames. The combined data derived from the exercise components and outdoor physical activity was aggregated to generate a physical activity report. The report allowed participants to monitor their physical activity behaviour and appeared on the “dashboard.” | 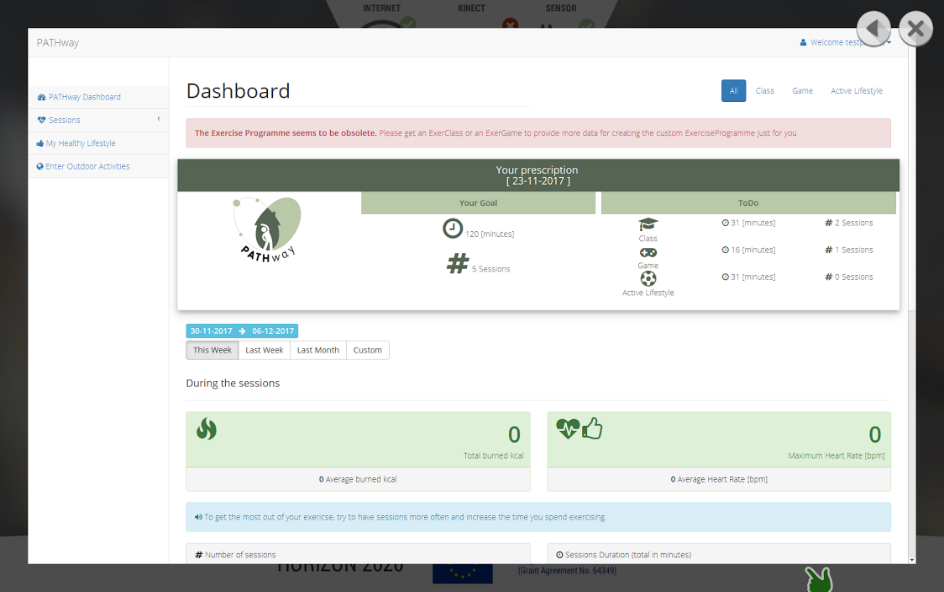 |
| Text messages | Participants received text messages with information on improving lifestyle-related risk factors for CVD. These text messages were tailored to the individual. Text messages were informed by the COM-B model and developed as part of the co-design of PATHway (12). Several lifestyle related cardiovascular risk factors were covered by PATHway -nutrition, stress, smoking, alcohol and medication adherence – and individuals identified if they wanted to make a change at the beginning of the trial. Automated motivational physical activity messages were also sent congratulating participants on their activity levels, encouraging them to become more active based on the activity recorded by the system. Where non-engagement with the PATHway system was identified, i.e. not logging of use, then messages prompting participants to engage were also sent. | 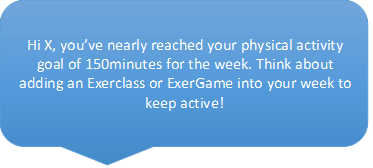 |
| Assessment | The assessment component allowed participants to assess their cardiovascular fitness by completing a two-minute step test. | 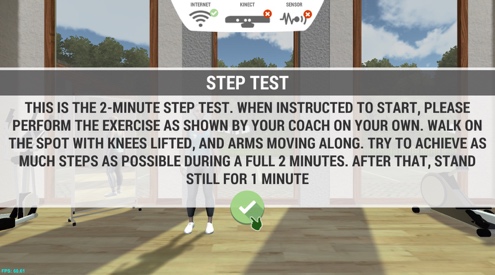 |
| ExerGame | The ExerGame provided participants the opportunity to engage in a game based exercise, the Microsoft Kinect sensor captured participants’ movements as they were required to perform certain exercises for example a squat to allow their game avatar to jump on logs so as to cross a river without falling into water. | 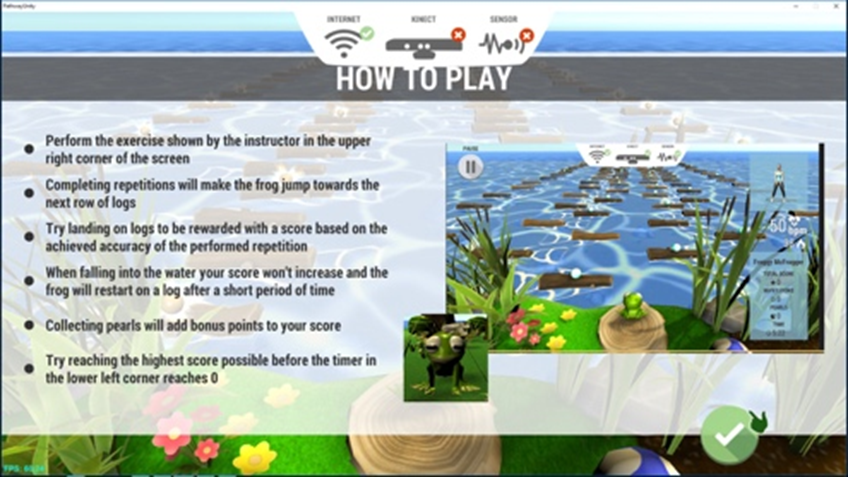 |
| Instructions | The instructions component contained detailed instructions on how to use the system. | **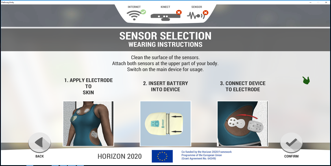** |
| Good Habits Visualisation | Key modifiable lifestyle behaviours for the management of CVD included physical activity, healthy eating, smoking cessation, alcohol moderation, stress management and medication adherence. Following a lifestyle assessment patients received, a personalised ‘Good Habits Visualisation’ of their own risk profile through PATHway. This ‘Good Habits Visualisation’ was based on the total scores from the lifestyle assessment completed by each individual at baseline. Their data was used to visualise how their current lifestyle fits in relation to CVD self-management guidelines. This visual graphic displays how each risk factor should be managed, through three simple categories: ‘Well Done’, ‘Room for Improvement’ and ‘Make a change’. The participant can then choose any of the behaviours to modify throughout the PATHway intervention. Good habits visualisation then leads the participant to an assessment whereby four questions are asked to ascertain the participant willingness to change that specific behaviour: *1) Is this behaviour a problem for you? 2) Are you distressed by this problem? 3) Are you interested in making a change? 4) Are you ready to change now?*. This assessment assigns the participant to a group appropriate to their behaviour change stage. Patients were deemed ‘ready for change [green category]’ if they had 4 yes answers, ‘ambivalent towards change’ [yellow category] if they had 2-3 yes answers and ‘not ready to change’ [red category] if they had 0-1 yes answers. For example those ready to change were set a goal and those who were deemed not ready were provided with education support. For more detailed information see (12). | 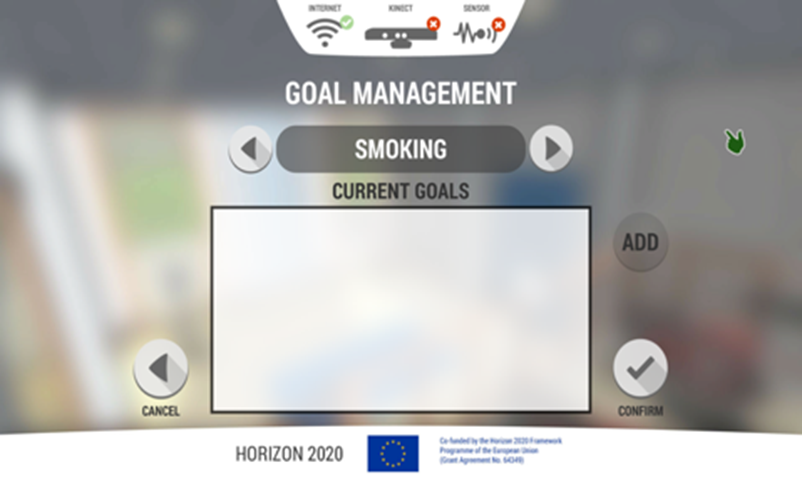 |
| Settings | The settings component enabled participants to alter the system to suit their needs, for example they could eliminate certain exercises from the ExerClass that were aggravated their comorbidities or that they found uncomfortable. | 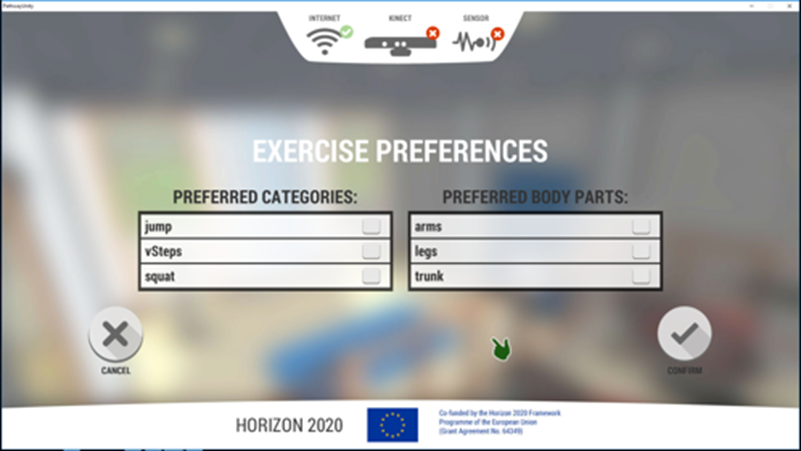 |
| Practice exercises | The practice exercises component allowed participants to isolate a certain exercise and follow detailed education information on how to conduct the exercise – the key teaching points – to practice this exercise, and also to learn how to adapt the exercise in order to make it more challenging (exercise progression) or less difficult (exercise adaptation). | 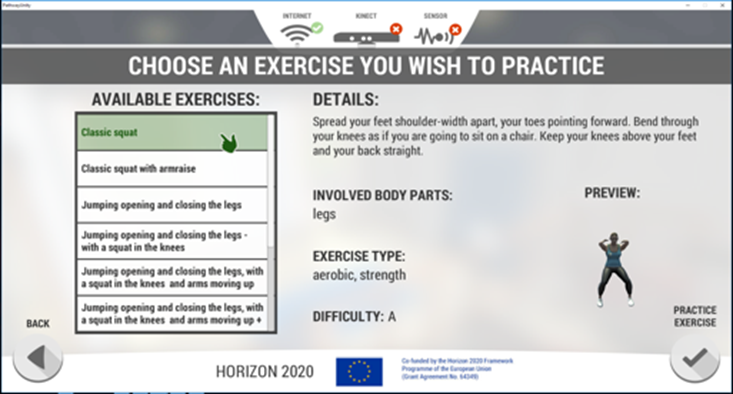 |
| Calendar/events | To facilitate social support within a community context, the calendar/events component enabled small groups of remote participants to exercise together by allowing them to communicate during the exercise session by means of headsets, as well as by sending messages and a live chat function. The calendar also allowed participants to promote events and to invite others to join, with a maximum of four participants per event. | 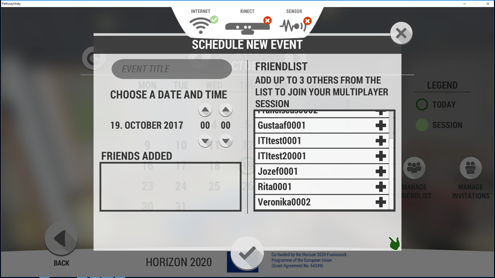 |

References

1. Triantafyllidis A, Filos D, Buys R, Claes J, Cornelissen V, Kouidi E, et al. Computerized decision support for beneficial home-based exercise rehabilitation in patients with cardiovascular disease. Comput Methods Programs Biomed. 2018;162:1-10.

2. Vanhees L, Geladas N, Hansen D, Kouidi E, Niebauer J, Reiner Z, et al. Importance of characteristics and modalities of physical activity and exercise in the management of cardiovascular health in individuals with cardiovascular risk factors: recommendations from the EACPR. Part II. Eur J Prev Cardiol. 2012;19(5):1005-33.
