# Supplementary material for: A qualitative exploration of cardiovascular disease patients’ views and experiences with an eHealth cardiac rehabilitation intervention: The PATHway Project
Source: PLoS One. 2020 Jul 6;15(7):e0235274. doi: 10.1371/journal.pone.0235274 (PMC7337342; doi:10.1371/journal.pone.0235274)
Supplement: Attachment — Submitted filename: Response to reviewers.docx [file pone.0235274.s003.docx]

Dear Reviewer,

Thank you for taking the time to review our revised manuscript and for your comments. We have made changes as recommended.

Kind regards,

Orlagh O’Shea

| Reviewers comments | Response |
| --- | --- |
| Reviewer #2: I have re-reviewed this manuscript, which has improved but I have some minor comments.  1) Page 4. I suggest to delete the new sentence “While quantitative data gives us information with regard to the effectiveness of the intervention it does not tell us whether the participants actually enjoyed or liked the intervention has been documented predictor of maintenance (10)”.  I think this sentence is not needed as the sentence before (regarding qualitative) has been clarified. | Thank you for this recommendation this sentence has been removed. |
| 2) Below is from our previous conversation: I suggest considering adding the participants levels of physical activity in table 1. These are presented in REF 10, but the analysis of the debriefs in the present study, may be reflected in light of PA levels (see further my comment 13). Information on civil status can also be added in table 1  Authors´ response Information on participants data captured by PATHway system was anonymized, therefore we do not have their physical activity levels during the intervention.  Additional comment: My suggestion was to add baseline PA levels and civil status in Table 2. | Baseline physical activity and civil status have been added to Table 2 |
| 3) Page 23 Referring to this sentence “At its core, the PATHway intervention is a behaviour change intervention...” Here it can be stated that the exercise platform is the core component in the PATHway system (to be in line with other changes performed in the method section). | In the methods PATHway is described as “a home-based, technology enabled complex behavior change intervention. It provides regular exercise sessions as the basis upon which to provide a personalized, comprehensive lifestyle intervention program to enable patients to self-manage their CVD and to lead a healthier lifestyle in general.” It was designed for the exercise to be the core component. A minor change has been made to this sentence (page 24, line 286-288):  *The PATHway intervention is a behaviour change intervention and specific behaviour change techniques were utilised including: self-monitoring, feedback, social support and individual tailoring (16)* |
| 4) Page 25. Regarding the following sentence in the discussion: “The perceived lack of need of the Good Habits Visualisation is potentially due to a lack of understanding or poor perception of CVD risk and not a lack of motivation to change as participants reported health as a motivation to engage with PATHway in the first instance”.  Additional comment: Can it be the case that professionals fails to meet patients as persons with individual preferences, needs and resources (not tailored for each person)?, and which may lead to a lack of understanding. I suggest to change/elaborate the sentence above. | Thank for you for this comment we have made changes to reflect the lack of personalisation in the group setting out cardiac rehabilitation. See page 25 line 325- 328.  *It could also be argued that the delivery of education in a group setting of cardiac rehabilitation during outpatient CR does not adequately meet each individuals’ needs and preferences, although previous research in diabetes patients has shown equal effectiveness between group and individual education (22).* |
